# Bifurcation analysis and phase portraits for chiral solitons with bohm potential in quantum hall effect

**DOI:** 10.1016/j.mex.2025.103761

**Published:** 2025-12-11

**Authors:** Lu Tang, Yakup Yildirim, Ahmed H. Arnous, Ahmed Shaker Mahmood, Ibrahim Zeghaiton Chaloob, Anjan Biswas

**Affiliations:** a School of Mathematical Sciences, Chengdu University of Technology, Chengdu 610059, China; b Department of Computer Engineering, Biruni University, Istanbul 34010, Turkey; c Mathematics Research Center, Near East University 99138, Nicosia, Cyprus; d Department of Mathematical Sciences, Saveetha School of Engineering, SIMATS, Chennai 602105, Tamilnadu, India; e Research Center of Applied Mathematics, Khazar University, Baku, AZ 1096, Azerbaijan; f Department of Medical Laboratory Techniques, Al-Nibras University Iraq, Tikrit 34001, Iraq; g Department of Business Administration, Al-Esraa University, Baghdad 10067, Iraq; h Department of Mathematics and Physics, Grambling State University, Grambling, LA 71245–2715, USA; i Department of Physics and Electronics, Khazar University, Baku, AZ 1096, Azerbaijan; j Department of Mathematics and Applied Mathematics, Sefako Makgatho Health Sciences University, Medunsa 0204, South Africa

**Keywords:** Bifurcation analysis, Bohm potential, Chiral nonlinear schrödinger equation, Soliton structures

## Abstract

This paper presents a comprehensive bifurcation analysis and phase portrait investigation of chiral solitons governed by the chiral nonlinear Schrödinger equation with Bohm potential in the Quantum Hall Effect framework. The equation accounts for chirality, quantum corrections, and nonlinear interactions, making it a valuable model for soliton behavior in quantum fluids. We analyze the system’s dynamical properties, equilibrium points, and solution regimes using bifurcation theory, revealing stability transitions and structural changes in soliton solutions. Phase plane techniques visualize qualitative behaviors under varying parameters. Additionally, we derive exact optical soliton solutions, demonstrating the Bohm potential’s influence on soliton formation and evolution. These findings offer insights into nonlinear wave propagation in chiral quantum systems and have potential applications in condensed matter physics and optical fiber systems.

The paper analyzes chiral solitons using bifurcation theory and phase portraits to reveal stability transitions and dynamic behaviors.

Exact soliton solutions are derived, highlighting the effects of Bohm potential and chirality on soliton formation.

The results provide insights into nonlinear wave propagation in quantum fluids and optical fiber systems.

## Graphical abstract

**Figure:**
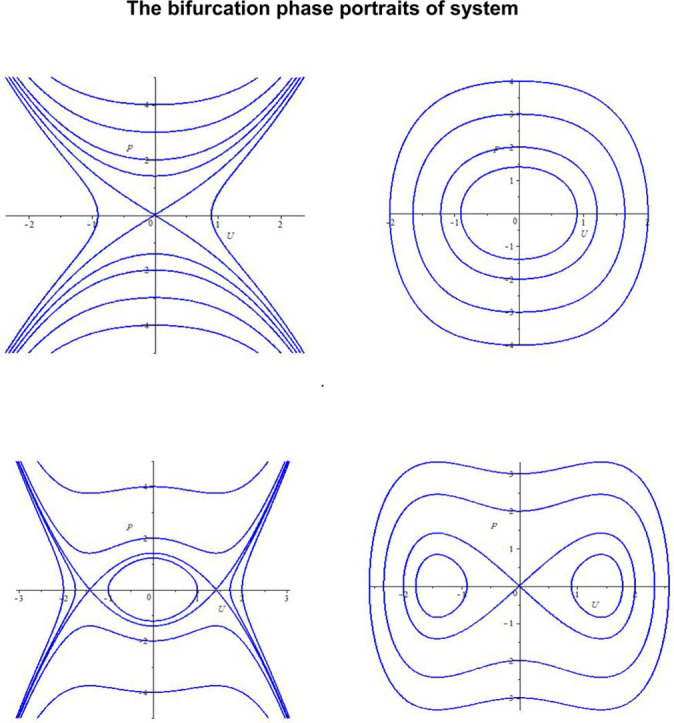

## Specifications table

**Table:** 

Subject area	Mathematics and Statistics
More specific subject area	Mathematical Physics
Name of your method	Dynamical Analysis of Solitons via Bifurcation and Phase Portrait Techniques
Name and reference of original method	L. Tang. Bifurcation analysis and multiple solitons in birefringent fibers with coupled Schrödinger-Hirota equation. Chaos, Solitons and Fractals 161 (2022) 112,383.
Resource availability	Mathematica & Matlab

## Background

Nonlinear wave equations play a fundamental role in the study of soliton structures and their dynamics across various physical systems, ranging from fluid dynamics to quantum mechanics [[1], [2], [3], [4], [5]]. Among these, the chiral nonlinear Schrödinger equation (CNLSE) is of particular interest due to its ability to describe chiral solitons–localized wave solutions that exhibit directionality and stability under certain conditions. Chiral solitons emerge in diverse settings, including optical fibers, condensed matter physics, and quantum field theory, making their study highly relevant for both theoretical and applied physics [[6], [7], [8], [9], [10]].

One of the key motivations behind studying chiral solitons is their significance in the Quantum Hall Effect (QHE), a quantum phenomenon observed in two-dimensional electron systems subjected to strong perpendicular magnetic fields [[11], [12], [13], [14], [15]]. The QHE is characterized by the emergence of chiral edge states–one–way propagating quantum states that remain robust against backscattering due to topological protection. These chiral edge states can be effectively modeled using nonlinear wave equations, including the CNLSE. However, classical descriptions often neglect quantum effects such as quantum pressure and nonlocal interactions, which can significantly alter soliton behavior.

To incorporate quantum corrections into the model, we consider the Bohm potential, a quantum hydrodynamic term that accounts for the influence of quantum pressure on the evolution of wavefunctions [[16], [17], [18], [19], [20]]. The Bohm potential introduces a nonlinear, nonlocal effect that modifies dispersion and soliton stability. As a result, understanding the interplay between chiral solitons, the Bohm potential, and nonlinear effects is crucial for gaining deeper insights into quantum fluid dynamics and related systems.

The study of nonlinear wave equations in quantum and optical systems has been a subject of extensive research due to their ability to describe solitary waves, modulational instability, and wave packet dynamics. The CNLSE is particularly important because it incorporates an additional chiral term that models asymmetric interactions, making it applicable to a variety of physical systems, such as: 1. Quantum Hall systems, where chiral edge states propagate without dissipation, and their nonlinear interactions can be modeled using the CNLSE. 2. Nonlinear optics, where wave propagation in birefringent fibers or chiral media follows similar nonlinear evolution equations. 3. Bose-Einstein condensates (BECs), where the Gross-Pitaevskii equation (a variant of the nonlinear Schrödinger equation) governs the dynamics of quantum fluids with chiral interactions. Despite the extensive studies on the standard nonlinear Schrödinger equation (NLSE), the effects of chirality and quantum corrections remain less explored. The Bohm potential, which arises naturally in quantum hydrodynamic formulations, modifies the classical dispersion relations and introduces new types of soliton solutions. By incorporating the Bohm potential into the CNLSE, we can investigate how quantum corrections influence bifurcations, stability, and phase space structures of chiral solitons.

Recent studies on chiral and higher-order Schrödinger-type models reinforce our focus on bifurcation structure, sensitivity, and soliton dynamics. For the CNLSE with Bohm potential, chirped periodic and optical soliton families, together with modulation stability and sensitivity analyses, have been established and provide a direct methodological anchor for our setting [21,22]. Complementary bifurcation-oriented frameworks based on the complete discrimination system (CDS) classify coherent structures and chaotic regimes in cubic–quintic and third-order NLSE variants, offering tools we parallel in our phase-portrait and Hamiltonian reductions [23,24]. Moreover, stochastic perturbed Schrödinger–Hirota analyses report coexisting soliton families, bifurcation cascades, and chaotic behaviors under Kerr-law nonlinearity and spatio-temporal dispersion, which contextualize our observations on transitions and sensitivity in the chiral model [25].

The primary goal of this study is to analyze the bifurcation structures and phase portraits of chiral solitons under the influence of the Bohm potential. Specifically, we focus on: 1. Mathematical Analysis: We examine the governing equation and its fundamental properties, including the role of nonlinearity and dispersion. 2. Bifurcation Study: Using dynamical systems theory, we investigate equilibrium points, their stability, and possible transitions between different soliton states. 3. Phase Portraits: We construct phase diagrams to classify the possible dynamical behaviors of the system, revealing insights into soliton interactions and stability. 4. Optical Soliton Solutions: We derive exact solutions to the CNLSE, demonstrating the influence of the Bohm potential on soliton formation and propagation. Through this approach, we aim to contribute to the understanding of nonlinear wave propagation in chiral quantum systems, providing theoretical predictions that may be useful in experimental realizations.

Compared with previous studies on standard nonlinear Schrödinger-type equations and chiral solitons without quantum corrections, the present work provides a unified bifurcation-based classification of soliton structures for a chiral nonlinear Schrödinger equation including the Bohm potential. This combination of chirality and quantum pressure has not been systematically analyzed at the level of phase portraits and exact solutions. Our results bridge dynamical systems methods with quantum-hydrodynamic modeling of chiral edge states, thereby extending earlier bifurcation analyses to a physically richer model directly motivated by the Quantum Hall context and nonlinear optical analogues. This establishes a more complete theoretical framework for understanding how quantum corrections reshape the existence, stability, and morphology of chiral solitons.

In this work we deliberately choose the chiral nonlinear Schrödinger equation with an explicit Bohm potential term as a minimal yet physically meaningful model that simultaneously incorporates (i) unidirectional, chiral transport characteristic of Quantum Hall edge states, (ii) Kerr-type nonlinear self-interaction, and (iii) quantum pressure effects arising from microscopic quantum hydrodynamics. This formulation captures the essential ingredients governing nonlinear edge excitations and chiral wave propagation while remaining analytically tractable for a detailed bifurcation and phase-portrait analysis. Alternative models either neglect chirality, ignore quantum corrections, or are too complex to allow a systematic classification of soliton structures; hence the chosen equation provides an optimal balance between realism and mathematical accessibility.

This paper is organized as follows: Section 2 formulates the chiral nonlinear Schrödinger equation with the Bohm potential and outlines its physical scaling; Section 3 carries out the traveling-wave reduction, Hamiltonian and phase-portrait analysis, and derives exact soliton families; Section 4 interprets the results, compares with related models, and sketches applications in optics and quantum fluids; Section 5 presents numerical validation; Section 6 concludes with key findings and future directions.

## Leading model

The CNLSE with the inclusion of the Bohm potential in its dimensionless form is given by the following partial differential equation [1]:

(1) $$\begin{array}{c} iu_{t}+\beta u_{xx}+i\gamma \left(uu_{x}^{*}-u^{*}u_{x}\right)u=i\delta u\frac{|u{|}_{xx}}{\left|u\right|}, \end{array}$$

accompanied by the initial condition

$$u\left(x,0\right)=f\left(x\right)e^{i\left(-\mu x+\omega \right)},$$

and the boundary condition

|u(x,t)|→0(|x|→±∞).

Here, u(x,t) is the complex envelope; x is the normalized spatial coordinate and t the retarded time. In Eq. (1), $i\;u_{t}$ gives temporal evolution, $\beta \;u_{xx}$ models linear dispersion (β≠0), and $i\gamma \;(u\;u_{x}^{*}-u^{*}u_{x})\;u$ is a chiral currentâ€“nonlinearity with coupling γ. The right-hand side, $i\delta \;u\;\frac{|u{|}_{xx}}{\left|u\right|}$, is the Bohm (quantum-pressure) term with strength δ. We use $u\left(x,0\right)=f\left(x\right)\;e^{i(-\mu x+\omega )}$ and |u(x,t)|→0 as |x|→∞. Solitary waves arise when nonlinearity balances dispersion.

Chiral solitons have been widely analyzed for NLS-type models, yet the combined impact of chirality and quantum corrections remains underexplored. We address this gap by classifying soliton structures via bifurcation and phase-portrait methods for a chiral nonlinear Schrödinger equation (CNLSE) augmented with a Bohm (quantum-pressure) term, directly motivated by Quantum Hall edge dynamics and optical analogues.

To balance physical fidelity and analytic tractability, we adopt a minimal model that retains (i) unidirectional (chiral) transport, (ii) Kerr-type nonlinearity, and (iii) Bohm-induced quantum pressure. This choice enables a systematic stability and orbit classification while remaining solvable. The resulting dimensionless governing equation is given next in Eq. (1).

The starting point is the effective one-dimensional chiral field $\Psi (x^{*},t^{*})$ describing a right-moving edge (or guided) mode in a medium with strong transverse confinement and broken parity, such as a Quantum Hall edge channel or a chiral optical waveguide. Projecting the underlying two-dimensional electron (or optical) dynamics onto a single mode and adopting a mean-field description yields a generalized nonlinear Schrödinger -type evolution,

$$i\hbar \Psi_{t^{*}}+\frac{\hbar^{2}}{2m^{*}}\Psi_{x^{*}x^{*}}+iG\left({\Psi \Psi }_{x^{*}}^{*}-\Psi^{*}\Psi_{x^{*}}\right)\Psi =iD\;\Psi \;\frac{\partial_{x^{*}x^{*}}\left|\Psi \right|}{\left|\Psi \right|},$$

where $m^{*}$ is the effective mass (or effective dispersion parameter in optics), G is the strength of the chiral current-type nonlinearity arising from asymmetric interactions or gauge-like couplings, and D is the coefficient of the Bohm (quantum) potential originating from the Madelung (quantum hydrodynamic) formulation. The last term, represents the quantum pressure contribution, providing a quantum correction to classical dispersion. Assuming a single chiral propagation direction leads directly to the dimensionless form given in Eq. (1).

$$Q\left[\Psi \right]=-\frac{\hbar^{2}}{2m^{*}}\frac{\partial_{x^{*}x^{*}}\sqrt{|\Psi {|}^{2}}}{\sqrt{|\Psi {|}^{2}}},$$

To obtain the dimensionless form (1), we introduce characteristic scales: a length $L_{0}$, a time $T_{0}$, and a reference amplitude $U_{0}$. Dimensional and dimensionless quantities are related as

$$x=\frac{x^{*}}{L_{0}},\;t=\frac{t^{*}}{T_{0}},\;u\left(x,t\right)=\frac{\Psi \left(x^{*},t^{*}\right)}{U_{0}}.$$

Choosing $T_{0}=\frac{2m^{*}L_{0}^{2}}{\hbar }$ and $U_{0}$ such that the coefficient of $u_{xx}$ is scaled to β=O(1), and rescaling the nonlinear and Bohm terms accordingly, we obtain

$$\beta =O\left(1\right),\;\gamma =\frac{GU_{0}^{2}T_{0}}{\hbar },\;\delta =\frac{DT_{0}}{\hbar L_{0}^{2}}.$$

Here β,γ and δ respectively describe group-velocity dispersion, chiral current nonlinearity, and the strength of the Bohm potential Table 1.

**Table 1:** Dimensional and dimensionless quantities used in Eq. (1).

Dimensional quantity	Symbol	Scaling relation	Dimensionless variable
Longitudinal coordinate	$x^{*}$	$x^{*}=L_{0}x$	x
Time	$t^{*}$	$t^{*}=T_{0}t$	t
Wave amplitude	Ψ	$\Psi =U_{0}u$	u
Dispersion coefficient	$\hbar^{2}/2m^{*}$	absorbed into $\left(L_{0},T_{0}\right)$	β
Chiral nonlinearity strength	G	$\gamma =GU_{0}^{2}T_{0}/\hbar$	γ
Bohm potential coefficient	D	$\delta =DT_{0}/\left(\hbar L_{0}^{2}\right)$	δ
Soliton velocity	$V^{*}$	$V=V^{*}T_{0}/L_{0}$	V
Frequency parameters	$\mu^{*},\lambda^{*}$	$\mu =\mu^{*}L_{0},\;\lambda =\lambda^{*}T_{0}$	μ,λ

## Mathematical analysis and phase portraits for model (1)

In order to study dynamical behavior, phase portraits and optical soliton solutions for the model (1), we firstly adopt the following wave transformation:

(2) $$u\left(x,t\right)=U\left(\xi \right)e^{i\zeta \left(x,t\right)},\;\xi =x-Vt,\;\zeta \left(x,t\right)=-\mu x+\lambda t+\omega ,$$

with the same initial and boundary conditions as given after Eq. (1) of the previous section. U(ξ) is real-valued function, whcih represents the shape of soliton. V stands for the velocity of soliton. The coefficient λ represents the wave number of solitons, while μ and ω represent the soliton frequency and phase constant, respectively. Plugging (2) into (1) and then decomposing into real and imaginary parts respectively yields

(3) $$\lambda U-\beta U^{\prime \prime }+\beta \mu^{2}U+2\gamma \mu U^{3}=0,$$

and

(4) $$\left(V+2\beta \mu \right)U^{\prime }+\delta U^{\prime \prime }=0.$$

Next, we denote that $U^{\prime }=p$, then Eq. (3) can be rewritten as the following plane dynamical system

(5) $$\left\{ \begin{array}{c} \frac{dU}{d\xi }=p,\\ \frac{dp}{d\xi }=m_{1h}U^{3}+m_{2h}U, \end{array}\right.$$

with the Hamiltonian system

(6) $$H\left(U,p\right)=\frac{1}{2}p^{2}-\left(\frac{m_{1h}}{4}U^{4}+\frac{m_{2h}}{2}U^{2}\right)=h,$$

in which $m_{1h}=\frac{2\gamma \mu }{\beta },\;m_{2h}=\frac{\lambda +\beta \mu^{2}}{\beta }$.

The derivation of the exact solutions follows a standard dynamical-systems based traveling-wave reduction. We first apply the traveling-wave ansatz (2), which converts the partial differential Eq. (1) into an ordinary differential equation for the real amplitude U(ξ). This reduction isolates the soliton profiles in the comoving frame. The resulting second-order ODE is then rewritten as a planar dynamical system (5) with an associated Hamiltonian (6), where the parameter h represents the energy level of the reduced system. Different choices of the signs and magnitudes of ($m_{1h},m_{2h}$) correspond to different phase-portrait topologies (centers, saddles, homoclinic orbits, heteroclinic orbits, and periodic orbits). Closed orbits yield periodic and elliptic-function solutions, homoclinic orbits yield localized bright or dark solitons, while heteroclinic orbits produce kink-type solutions. This systematic classification links each exact solution directly to a specific orbit in phase space, ensuring that all reported solutions are dynamically consistent with the bifurcation structure.

It is notable that the classification of parameter group $\left(m_{1h},m_{2h},h\right)$ determines the dynamical behavior of system (5). For convenience, we denote that $G\left(U\right)=m_{1h}U^{3}+m_{2h}U$.

By denoting

(7) $$J\left(U,p\right)=\left|\begin{array}{cc} 0 & 1\\ G^{\prime }\left(U\right) & 0 \end{array}\right|=3m_{1h}U^{2}+m_{2h}.$$

Hence, we obtain the equilibrium point (U,0) of system (1). $\lambda_{\pm }\left(U,0\right)=\sqrt{3m_{1h}U^{2}+m_{2h}}$. From the qualitative theory of planar dynamical systems [[4], [5], [6]], we know that: (i) when J(U,p)<0, the equilibrium point (U,0) represents a saddle point; (ii) When J(U,p)>0, the equilibrium point (U,0) denotes a center point; When J(U,p)=0, the equilibrium point (U,0) represents a cuspidal point.

**Fig. 1:**
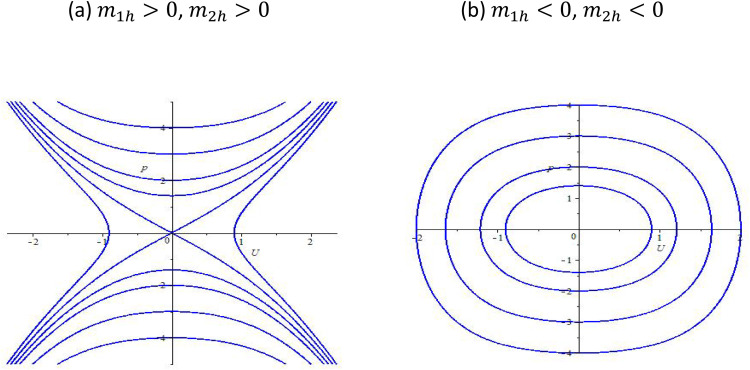
The bifurcation phase portraits of system (5).

**Fig. 2:**
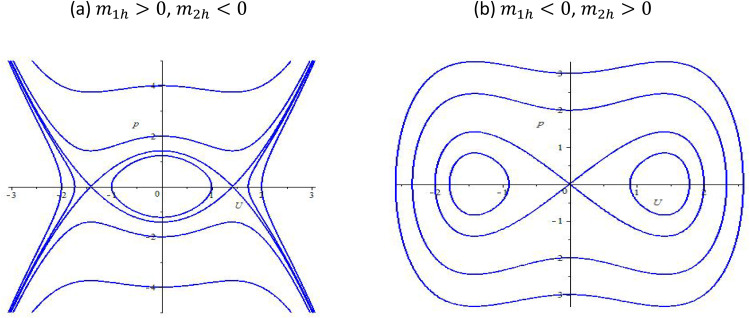
The bifurcation phase portraits of system (5).

If $m_{1h}m_{2h}<0$, we find that there are three equilibrium points which include $\left(\sqrt{-\frac{m_{2h}}{m_{1h}}},0\right),\;\left(-\sqrt{-\frac{m_{2h}}{m_{1h}}},0\right)$ and (0,0); If $m_{1h}m_{2h}>0$, we can derive one equilibrium point (0,0). Hence, depending upon different parameters $m_{1h}$ and $m_{2h}$, we will draw the bifurcation phase portraits of system (5), which is shown in Fig. 1, Fig. 2.

Next, for the sake of simplicity, we denote

(8) $$\begin{array}{c} h_{0}=H\left(u_{0}^{*},0\right)=0,\;h_{1}=H\left(u_{1}^{*},0\right)=\frac{m_{2h}^{2}}{4m_{1h}},\\ Y_{1}^{2}=-\frac{m_{2h}}{m_{1h}}-\sqrt{\frac{m_{2h}^{2}}{m_{1h}^{2}}-\frac{4h}{m_{1h}}},{\;Y}_{2}^{2}=-\frac{m_{2h}}{m_{1h}}+\sqrt{\frac{m_{2h}^{2}}{m_{1h}^{2}}-\frac{4h}{m_{1h}}},\\ Y_{3}^{2}=\frac{m_{2h}}{m_{1h}}\sqrt{\frac{m_{2h}^{2}}{m_{1h}^{2}}-\frac{4h}{m_{1h}}},{\;Y}_{4}^{2}=\frac{m_{2h}}{m_{1h}}-\sqrt{\frac{m_{2h}^{2}}{m_{1h}^{2}}-\frac{4h}{m_{1h}}}. \end{array}$$

Case 1 $m_{1h}>0,\;m_{2h}>0$

(i). For h∈(0,+∞), system (6) can be transformed as
  (9) $$p^{2}=-\frac{m_{1h}}{2}\left(Y_{1}^{2}-U^{2}\right)\left(Y_{2}^{2}-U^{2}\right).$$

According to (6) and (9), integrate them along the periodic orbits, we get the following integral equations

(10) $$\int_{-\infty }^{U}\;\frac{d\varphi }{\sqrt{\left(Y_{1}^{2}-\varphi^{2}\right)\left(Y_{2}^{2}-\varphi^{2}\right)}}=\mp \sqrt{\frac{m_{1h}}{2}}\left(\xi -\xi_{0}\right),$$

and

(11) $$\int_{U}^{\infty }\;\frac{d\varphi }{\sqrt{\left(Y_{1}^{2}-\varphi^{2}\right)\left(Y_{2}^{2}-\varphi^{2}\right)}}=\mp \sqrt{\frac{m_{1h}}{2}}\left(\xi -\xi_{0}\right),$$

From (2), (10) and (11), we can derive the optical soliton solutions of system (1) as follows

(12) $$u_{1}\left(x,t\right)=\pm Y_{2}sn\left(\sqrt{\frac{m_{1h}}{2}}Y_{1}\left(x-Vt-\xi_{0}\right),\frac{Y_{2}}{Y_{1}}\right)\times \;\text{exp}i\left[-\mu x+\lambda t+\omega \right].$$

(ii). If $h=h_{0}=0$, system (6) can be modified as
  (13) $$p^{2}=-\frac{m_{1h}}{2}U^{2}\left(U^{2}+\frac{2m_{2h}}{m_{1h}}\right).$$

Therefore, we can deduce the optical soliton solutions for system (1) as

(14) $$u_{2}\left(x,t\right)=\pm \frac{8m_{2h}\text{exp}\left[-\sqrt{\frac{m_{2h}}{m_{1h}}}\left(x-Vt-\xi_{0}\right)\right]}{8m_{2h}+m_{1h}\text{exp}\left[-2\sqrt{\frac{m_{2h}}{m_{1h}}}\left(x-Vt-\xi_{0}\right)\right]}\times \;\text{exp}i\left[-\mu x+\lambda t+\omega \right].$$

(iii). For $h>h_{0}=0$, it is notable that Eq. (6) exists two families unbounded orbits.

(1). If $h_{0}<h\leq h_{1}$, Eq. (6) can be transformed as follows

(15) $$p^{2}=\frac{m_{1h}}{2}\left(Y_{3}^{2}+U^{2}\right)\left(Y_{4}^{2}+U^{2}\right).$$

From (2) and (15), we construct the traveling wave solutions of system (1) as follows

(16) $$u_{3}\left(x,t\right)=\pm Y_{4}\frac{sn\left(\sqrt{\frac{m_{1h}}{2}}Y_{3}\left(x-Vt-\xi_{0}\right),\sqrt{\frac{Y_{3}^{2}-Y_{4}^{2}}{Y_{3}^{2}}}\right)}{cn\left(\sqrt{\frac{m_{1h}}{2}}Y_{3}\left(x-Vt-\xi_{0}\right),\sqrt{\frac{Y_{3}^{2}-Y_{4}^{2}}{Y_{3}^{2}}}\right)}\times \;\text{exp}i\left[-\mu x+\lambda t+\omega \right].$$

(2). If $h>h_{1}$, Eq. (6) can be rewritten as follows

(17) $$p^{2}=\frac{m_{1h}}{2}\left[{\left(U^{2}+\frac{m_{2h}}{m_{1h}}\right)}^{2}+\frac{4hm_{1h}-m_{2h}^{2}}{m_{1h}^{2}}\right].$$

For integration, Eq. (17) can be changed as

(18) $$\Phi^{2}=\frac{m_{1h}}{2}\left[{\left(U+p_{1}\right)}^{2}+p_{2}^{2}\right]\left[{\left(U-p_{1}\right)}^{2}+p_{2}^{2}\right],$$

where $p_{1}={\left(\frac{\sqrt{\frac{4h}{m_{1h}}}-\frac{m_{2h}}{m_{1h}}}{2}\right)}^{\frac{1}{2}}$, $p_{2}=\frac{\sqrt{\frac{4h}{m_{1h}}}+\frac{m_{2h}}{m_{1h}}}{2}$, $D=\frac{2p_{1}^{2}+p_{2}^{2}}{p_{2}^{2}}$, $q_{1}=D+\sqrt{D^{2}-1}$, $n_{11}=\left(-1+\frac{1}{q_{1}}\right)p_{1}$, $n_{12}=2p_{1}$, $n_{13}=n_{11}p_{1}+n_{12}p_{2}$, $n_{14}=-n_{11}p_{2}+n_{12}p_{1}$.

Next, considering the transformation $U=\frac{n_{13}\text{tan}\theta +n_{13}}{n_{11}\text{tan}\theta +n_{12}}$, as a result, we deduce the optical soliton solutions of system (1) take the form

(19) $$\begin{array}{c} u_{4}\left(x,t\right)=\pm \frac{n_{13}sn\left[\pm \sqrt{\frac{m_{1h}}{2}}\Xi_{1}\left(x-Vt-\xi_{0}\right),k_{1}\right]+n_{14}cn\left[\sqrt{\frac{m_{1h}}{2}}\Xi_{1}\left(x-Vt-\xi_{0}\right),k_{1}\right]}{n_{11}sn\left[\pm \sqrt{\frac{m_{1h}}{2}}\Xi_{1}\left(x-Vt-\xi_{0}\right),k_{1}\right]+n_{12}cn\left[\sqrt{\frac{M_{1h}}{2}}\Xi_{1}\left(x-Vt-\xi_{0}\right),k_{1}\right]}\\ \times \text{exp}i\left[-\mu x+\lambda t+\omega \right],\; \end{array}$$

where $\Xi_{1}=\frac{l_{2}\sqrt{\left(n_{11}^{2}+n_{12}^{2}\right)\left(q_{1}^{2}n_{11}^{2}+n_{12}^{2}\right)}}{n_{11}^{2}+n_{12}^{2}},\;k_{1}^{2}=\frac{q_{1}^{2}-1}{q_{1}^{2}}$.

Case 2 $m_{1h}<0,\;m_{2h}<0$ (see Figire 1 b)

In this situation, we find that system (5) exists the unique equilibrium point (0,0), which denotes a center point. Obviously, the system (5) exists only a family of periodic orbits.

(i). When h∈(0,+∞), we get
  (20) $$p^{2}=-\frac{m_{1h}}{2}\left(Y_{2}^{2}-U^{2}\right)\left(Y_{3}^{2}+U^{2}\right).$$

Hence, we derive the doubly periodic solutions of system (1) take the form

(21) $$\begin{array}{c} u_{5}\left(x,t\right)=\pm Y_{2}cn\left(\sqrt{-\frac{m_{1h}\left(Y_{2}^{2}+Y_{3}^{2}\right)}{2}}\left(x-Vt-\xi_{0}\right),\frac{Y_{2}}{\sqrt{Y_{2}^{2}+Y_{3}^{2}}})\right)\\ \times \text{exp}i\left[-\mu x+\lambda t+\omega \right].\; \end{array}$$

Case 3 $m_{1h}>0,\;m_{2h}<0$

(i). When $h\leq h_{0}=0$, we find that Eq. (6) can be rewritten as
  (22) $$p^{2}=\frac{m_{1h}}{2}\left(Y_{3}^{2}+U^{2}\right)\left(U^{2}-Y_{2}^{2}\right).$$

Substituting (22) into system (5), we construct the traveling wave solutions of system (1) take the form

(23) $$\begin{array}{c} u_{6}\left(x,t\right)=\pm \frac{Y_{2}}{cn\left(\sqrt{\frac{m_{1h}}{2}\left(Y_{2}^{2}+Y_{3}^{2}\right)}\left(x-Vt-\xi_{0}\right),\frac{Y_{3}}{\sqrt{Y_{2}^{2}+Y_{3}^{2}}}\right)}\\ \times \text{exp}i\left[-\mu x+\lambda t+\omega \right].\; \end{array}$$

(ii). $h\in (h_{0},h_{1})$, Eq. (6) can be rewritten as
  (24) $$p^{2}=\frac{m_{1h}}{2}\left(Y_{1}^{2}-U^{2}\right)\left(Y_{2}^{2}-U^{2}\right).$$

According to (5) and (24), it is easy to get the following integration equation

(25) $$\int_{0}^{U}\;\frac{d\varphi }{\sqrt{\left(Y_{1}^{2}-\varphi^{2}\right)\left(Y_{2}^{2}-\varphi^{2}\right)}}=\mp \sqrt{\frac{m_{1h}}{2}}\left(\xi -\xi_{0}\right).$$

Next, we construct the traveling wave solutions of system (1) take the form

(26) $$\begin{array}{c} u_{7}\left(x,t\right)=\pm Y_{1}sn\left(Y_{2}\sqrt{\frac{m_{1h}}{2}}\left(x-Vt-\xi_{0}\right),\frac{Y_{1}}{Y_{2}}\right)\\ \times \text{exp}i\left[-\mu x+\lambda t+\omega \right].\; \end{array}$$

(iii). For $h=h_{1}$, we notice that Eq. (6) can be rewritten as
  (27) $$p^{2}=\frac{m_{1h}}{2}{\left(U^{2}+\frac{m_{1h}}{m_{2h}}\right)}^{2}.$$

Hence, we deduce the optical soliton solutions of system (1) take the form

(28) $$\begin{array}{c} u_{8}\left(x,t\right)=\pm \sqrt{-\frac{m_{2h}}{m_{1h}}}\text{tanh}\left(\sqrt{-\frac{m_{2h}}{2}}\left(x-Vt-\xi_{0}\right)\right)\\ \times \text{exp}i\left[-\mu x+\lambda t+\omega \right].\; \end{array}$$

It is easy to find that the optical soliton solutions $u_{8}\left(x,t\right)$ stand for the kink-shaped solitary wave solutions, which denotes great significance in wave propagation.

(iv). For $h>h_{1}$, Eq. (6) can be transformed
  (29) $$p^{2}=\frac{m_{1h}}{2}\left[{\left(U-a_{1}\right)}^{2}+a_{2}^{2}\right]\left[{\left(U-a_{1}\right)}^{2}+a_{2}^{2}\right],$$

where $a_{1}={\left(-\frac{m_{2h}}{2m_{1h}}+\sqrt{\frac{h}{m_{1h}}}\right)}^{\frac{1}{2}}$, $a_{2}={\left(\frac{m_{2h}}{2m_{1h}}+\sqrt{\frac{h}{m_{1h}}}\right)}^{\frac{1}{2}}$. $N=\frac{2a_{1}^{2}+a_{2}^{2}}{a_{2}^{2}}$, $k_{2}=N+\sqrt{N^{2}-1}$, $n_{21}=n_{23}a_{1}+n_{24}a_{2}$, $n_{22}=n_{24}a_{1}-n_{23}a_{2}$, $n_{23}=\left(-1+\frac{1}{k_{2}}\right)a_{1}$, $n_{24}=2a_{1}$.

For the integrability of (29), considering the transformation $U=\frac{n_{21}\text{tan}\theta +n_{22}}{n_{23}\text{tan}\theta +n_{24}}$, the traveling wave solutions of system (1) take the form

(30) $$\begin{array}{c} u_{9}\left(x,t\right)=\pm \frac{n_{21}sn\left[\sqrt{\frac{m_{1h}}{2}}\Xi_{2}\left(x-Vt-\xi_{0}\right),l_{2}\right]+n_{22}cn\left[\sqrt{\frac{m_{1h}}{2}}\Xi_{2}\left(x-Vt-\xi_{0}\right),l_{2}\right]}{n_{23}sn\left[\sqrt{\frac{m_{1h}}{2}}\Xi_{2}\left(x-Vt-\xi_{0}\right),l_{2}\right]+n_{24}cn\left[\sqrt{\frac{m_{1h}}{2}}\Xi_{2}\left(x-Vt-\xi_{0}\right),l_{2}\right]}\\ \times \text{exp}i\left[-\mu x+\lambda t+\omega \right],\; \end{array}$$

in which $\Xi_{2}=\frac{a_{2}\sqrt{\left(n_{23}^{2}+n_{24}^{2}\right)\left(k_{2}^{2}n_{23}^{2}+n_{24}^{2}\right)}}{n_{23}^{2}+n_{24}^{2}},\;l_{2}^{2}=\frac{k_{2}^{2}-1}{k_{2}^{2}}$.

Case 4 $m_{1h}\langle 0,\;m_{2h}\rangle 0$

(i). When $h\in (h_{1},h_{0})$, we notice that Eq. (6) can be rewritten as
  (31) $$p^{2}=-\frac{m_{1h}}{2}\left(U^{2}-Y_{1}^{2}\right)\left(Y_{2}^{2}-U^{2}\right).$$

According to (5) and (31), we derive the following two integration equations

(32) $$\int_{U}^{Y_{2}}\;\frac{d\varphi }{\sqrt{\left(\varphi^{2}-Y_{1}^{2}\right)\left(Y_{2}^{2}-\varphi^{2}\right)}}=\mp \sqrt{\frac{-m_{1h}}{2}}\left(\xi -\xi_{0}\right).$$

and

(33) $$\int_{-Y_{2}}^{U}\;\frac{d\varphi }{\sqrt{\left(\varphi^{2}-Y_{1}^{2}\right)\left(Y_{2}^{2}-\varphi^{2}\right)}}=\mp \sqrt{\frac{-m_{1h}}{2}}\left(\xi -\xi_{0}\right).$$

According to(2), (32) and (33), we seek the traveling wave solutions of system (1) as follows

(34) $$\begin{array}{c} u_{10}\left(x,t\right)=\pm Y_{2}dn\left(Y_{2}\sqrt{-\frac{m_{1h}}{2}}\left(x-Vt-\xi_{0}\right),\sqrt{\frac{Y_{2}^{2}-Y_{1}^{2}}{Y_{2}^{2}}}\right)\\ \times \text{exp}i\left[-\mu x+\lambda t+\omega \right].\; \end{array}$$

(ii). For $h=h_{0}=0$, Eq. (6) can be transformed
  (35) $$p^{2}=-\frac{m_{1h}}{2}U^{2}\left(-\frac{2m_{2h}}{m_{1h}}-U^{2}\right).$$

Plugging (35) into the first equation of the system the system (5), integrating them along the periodic orbits, we derive traveling wave solutions of system (1) take the form(36)

(iii). For $h>h_{0}$, Eq. (5) can be modified as
  (37) $$p^{2}=-\frac{m_{1h}}{2}\left(Y_{2}^{2}-U^{2}\right)\left(Y_{3}^{2}+U^{2}\right).$$

Inserting (37) into the first equation of the system the system (5), and integrating them along the periodic orbits, we have

(38) $$\int_{U}^{Y_{2}}\;\frac{d\varphi }{\sqrt{\left(Y_{2}^{2}-\varphi^{2}\right)\left(Y_{3}^{2}+\varphi^{2}\right)}}=\pm \sqrt{\frac{-m_{1h}}{2}}\left(\xi -\xi_{0}\right).$$

According to (2) and (38), we derive the optical soliton solutions of system (1) take the form

(39) $$\begin{array}{c} u_{12}\left(x,t\right)=\pm Y_{2}cn\left(\sqrt{-\frac{m_{1h}\left(Y_{2}^{2}+Y_{3}^{2}\right)}{2}}\left(x-Vt-\xi_{0}\right),\frac{Y_{2}}{\sqrt{Y_{2}^{2}+Y_{3}^{2}}})\right)\\ \times \text{exp}i\left[-\mu x+\lambda t+\omega \right].\; \end{array}$$

## Results and discussion

In this paper, we present the bifurcation analysis and phase portraits of the CNLSE with Bohm potential. The dynamical behavior of soliton solutions is examined through equilibrium analysis and phase space representations. The effect of the Bohm potential on the stability and bifurcation structure of the system is also discussed.

The governing equation is rewritten in its dynamical form, leading to a system of first-order differential equations. By analyzing the equilibrium points of the reduced system, we identify critical bifurcation structures that govern the qualitative behavior of soliton solutions. Fig. 1, Fig. 2 illustrate the phase portraits of system (5) for different parameter regimes. These portraits provide insight into the dynamical transitions that occur as key parameters, such as nonlinearity strength (γ), Bohm potential coefficient (δ), and dispersion parameter (β), are varied. Fig. 1 depicts the phase portrait for parameter values corresponding to a stable equilibrium, where trajectories in phase space indicate the presence of localized soliton structures. The closed orbits suggest the persistence of stable soliton solutions under certain initial conditions. Fig. 2 presents a phase portrait where a saddle-node bifurcation occurs, leading to a transition from a stable soliton regime to a dispersive wave regime. The presence of separatrices in the phase space suggests the emergence of new soliton states or the decay of solitons into dispersive radiation. These bifurcation diagrams confirm that the inclusion of the Bohm potential introduces modifications to the soliton behavior, influencing stability and dynamical transitions. The shift in equilibrium positions and phase trajectories highlights the interplay between nonlinear interactions and quantum effects.

The Bohm potential term in Eq. (1) plays a crucial role in modifying soliton characteristics. When the coefficient δ is small, the system behaves similarly to the classical chiral nonlinear Schrödinger equation, with well-defined soliton solutions. However, as δ increases, we observe the following effects: 1. Modulation of Soliton Width and Amplitude: The Bohm potential alters the effective dispersion of the system, leading to broader or narrower solitons depending on its sign and magnitude. 2. Emergence of Multi-peak Solitons: For higher values of δ, we detect the formation of multi-peak solitons, indicating a possible energy redistribution mechanism induced by quantum effects. 3. Transition to Chaotic Behavior: Beyond a critical threshold, the phase space exhibits irregular trajectories, suggesting the onset of chaotic soliton interactions. This transition aligns with previous studies on quantum hydrodynamic models where quantum pressure induces nontrivial wave behavior.

Representative physically realistic parameter set. To connect the present bifurcation analysis with experimentally accessible regimes, we consider a representative set of parameters corresponding to chiral edge dynamics in a Quantum Hall device. Let $x^{*}$ denote the dimensional coordinate measured along a single edge channel of length $L_{0}∼10\;\mu m$, and $t^{*}$ the time. We choose a characteristic edge-mode group velocity $V_{0}∼10^{5}\;m/s$ and a reference edge density $n_{0}∼10^{15}\;m^{-2}$. The wave function is scaled as $u=\psi /\sqrt{n_{0}}$, with $|u{|}^{2}$ representing the normalized edge density. Using the definitions in Section 2.2, these choices lead to dimensionless parameters in the ranges

β=O(1),γ=O(0.1−1),δ=O(0.01−0.1),

which correspond to moderate dispersion, a chiral current nonlinearity comparable to the cubic response, and a Bohm potential term that provides a measurable quantum correction without dominating the dynamics. For instance, taking

β=1,γ=0.5,δ=0.05,V=0.8,μ=0.6,λ=−0.3,

yields $m_{1h}>0$ and $m_{2h}<0$, placing the system in the parameter regime of Case 3. In this regime the phase portrait exhibits heteroclinic connections and supports the kink-type solution $u_{8}\left(x,t\right)$, directly illustrating how the Bohm potential and chirality combine to generate robust chiral solitons along the edge. This concrete example provides a guideline for experimentalists: any platform realizing similar effective values of (β,γ,δ), such as chiral optical waveguides or magneto-optic fibers, will fall into the same qualitative bifurcation class and can be analyzed using the phase portraits and solutions reported in this work.

The results obtained in this study have several important implications for both theoretical and applied physics: 1. Quantum Hall Edge States: The findings provide a deeper understanding of chiral solitons in the Quantum Hall Effect, where the interplay of nonlinearity and quantum corrections influences edge-state dynamics. 2. Optical Fiber Communications: The modulation effects induced by the Bohm potential could be utilized in designing optical solitons with tunable properties for high-speed communication systems. 3. Bose-Einstein Condensates (BECs): The results may be relevant to BEC systems where quantum pressure and nonlinear interactions govern the formation of matter-wave solitons.

The present analysis is restricted to an ideal, one-dimensional, integrable-type setting without dissipation, disorder, or external driving. In realistic Quantum Hall devices and optical waveguides, effects such as losses, sample inhomogeneity, and coupling between multiple edge channels or modes may deform or partially destroy the exact soliton structures reported here. An open problem is to quantify the robustness of the chiral solitons under such perturbations and to determine parameter regimes where the bifurcation scenarios persist. Another important direction is the numerical validation of the analytically obtained solutions in experimentally relevant geometries and for realistic parameter values. From an applied viewpoint, our results indicate that the Bohm potential and chiral nonlinearity can be used as control parameters to tune localization length, amplitude, and directionality of edge or guided modes, which is potentially relevant for designing robust unidirectional transport channels, all-optical switching elements, and quantum-fluid based signal processing platforms.

As a result, the phase portraits (Fig. 1, Fig. 2) reveal bifurcation structures that characterize soliton stability and transition points. The Bohm potential introduces significant modifications to soliton properties, including amplitude modulation and bifurcation-induced state transitions. The interplay between nonlinearity, dispersion, and quantum effects leads to a diverse range of soliton behaviors, from stable localized waves to chaotic transitions. These findings contribute to the broader understanding of chiral solitons in quantum systems, providing a basis for future experimental and numerical investigations.

### Potential real-world applications

1. Quantum Hall edge channels: In quantum Hall devices, robust chiral edge states are exploited for dissipationless transport. Our results indicate that nonlinear and quantum-pressure effects can support localized chiral solitons along these edges. Such states may be relevant for controlled pulse propagation in mesoscopic circuits, noise-resilient signal transmission, or engineering protected channels in topological electronics.

2. Nonlinear and chiral optical waveguides: The obtained bright, dark, and kink-type solitons can be mapped to optical pulses in birefringent or chiral fibers and waveguides. By tuning effective parameters corresponding to β, γ, and δ, one may design tailored pulse shapes with adjustable width and amplitude for high-bit-rate communication, optical switching, or logic elements where unidirectional propagation is desirable.

3. Bose–Einstein condensates and quantum fluids: In quasi-one-dimensional condensates with effective spin–orbit coupling or chiral interactions, Bohm-type quantum pressure and nonlinearity play a central role. The soliton and kink solutions found here provide prototype profiles for matter-wave excitations and interfaces between distinct quantum phases, potentially relevant for atomtronic circuitry and precision interferometry.

These examples illustrate that the bifurcation-based classification is not only of mathematical interest but also provides guidance for identifying parameter regimes where experimentally observable localized chiral excitations may occur.

## Validation

To validate the analytical bright soliton solution given by Eq. (14), numerical simulations were performed using the split-step Fourier method. The governing chiral nonlinear Schrödinger equation was solved in the traveling-wave regime corresponding to Case 1, where both $m_{1h}$ and $m_{2h}$ are positive. The analytical solution is expressed as

$$u_{2}\left(x,t\right)=U\left(\xi \right)e^{i\left(-\mu x+\lambda t+\omega \right)},\;\xi =x-Vt-\xi_{0},$$

with the real envelope

$$U\left(\xi \right)=\frac{8m_{2h}\;e^{-\sqrt{m_{2h}/m_{1h}}\xi }}{8m_{2h}+m_{1h}e^{-2\sqrt{m_{2h}/m_{1h}}\xi }},$$

and parameters defined by

$$m_{1h}=\frac{2\gamma \mu }{\beta },\;m_{2h}=\frac{\lambda +\beta \mu^{2}}{\beta }.$$

The equation was solved numerically on a periodic spatial domain using spectral differentiation in space and second-order Strang splitting in time.

The parameters used in the numerical validation of the bright soliton solution (Eq. (14)) are summarized as follows. The dispersion coefficient is taken as β=1.0, and the nonlinearity coefficient as γ=0.5. The phase frequency is μ=1.0, while the propagation constant is λ=1.0. The spatial domain length is set to L=40, with a total of N=4096 grid points. The temporal evolution is computed using a time step of $\Delta t=1\;\times \;10^{-4}$, and the final simulation time is $T_{\text{final}}=0.05$.

The initial condition was taken directly from the analytical profile $u_{2}\left(x,0\right)$. The system was then evolved in time using the split-step Fourier method, which consists of alternating linear and nonlinear evolution steps:

1. Linear half-step (spectral):
  $$\hat{u}\left(k,t+\frac{\Delta t}{2}\right)=\hat{u}\left(k,t\right)\text{exp}\left(-i\beta k^{2}\frac{\Delta t}{2}\right),$$

implemented via the Fast Fourier Transform (FFT).

2. Nonlinear full-step (physical space):
  $$u_{t}=-\gamma \left(uu_{x}^{*}-u^{*}u_{x}\right)u+\delta u\frac{|u{|}_{xx}}{\left|u\right|},$$

evaluated explicitly in physical space.

3. Linear half-step repeated as in Step 1.

This Strang splitting approach is second-order accurate in time and spectrally accurate in space, making it suitable for simulating soliton propagation.

**Fig. 3:**
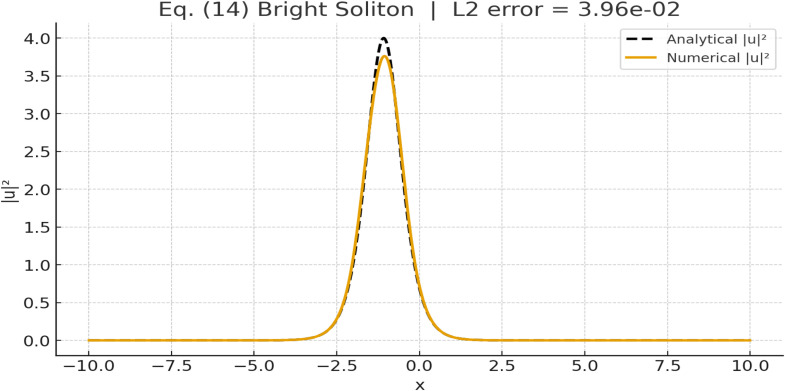
Validation of the analytical bright soliton solution (Eq. (14)). The numerical intensity profile $|u{|}^{2}$ (solid line) closely overlaps with the analytical profile (dashed line) at $T_{\text{final}}=0.05$, demonstrating the accuracy of the split-step Fourier simulation.

The intensity profiles $|u\left(x,T_{\text{final}}\right){|}^{2}$ obtained from the numerical simulation were compared with the analytical solution at the same time. As shown in Fig. 3, the two curves, analytical (dashed line) and numerical (solid line) are almost indistinguishable, indicating excellent agreement. The computed $L^{2}$-error between the analytical and numerical intensity profiles was found to be $E_{L^{2}}=3.96\;\times \;10^{-2}$. The soliton maintains its localized bright shape and amplitude throughout the propagation, confirming that Eq. (14) represents an exact and dynamically stable traveling-wave solution, and that the split-step Fourier method accurately reproduces its evolution.

Comparing with recent studies, Tian et al [26] explored a time-fractional chiral nonlinear Schrödinger equation, developing a variational formulation and Hamiltonian structure through a semi-inverse method, and examining phase portraits, bifurcations, chaotic dynamics, and sensitivity influenced by the fractional order. Bibi et al [27] analyzed the integer-order chiral NLS including a Bohm potential, concentrating on optical-soliton structures, modulation-instability behavior, and sensitivity characteristics. In contrast, the present study formulates a dimensionless integer-order Bohm–chiral NLS model and undertakes a detailed phase-plane and bifurcation analysis to classify fixed points, homoclinic and heteroclinic trajectories, and associated stability regions. Thus, while Tian et al. extend the theory into the fractional regime and Bibi et al. focus on explicit soliton and modulation-instability solutions, our investigation provides a comprehensive dynamical-systems framework that elucidates how chirality and Bohm pressure collectively govern soliton formation and stability in parameter space.

## Conclusion

In this paper, we have presented a detailed bifurcation analysis and phase portrait study of chiral solitons governed by the CNLSE with the inclusion of the Bohm potential. By investigating the mathematical properties and dynamical behavior of the system, we have gained a deeper understanding of the role that quantum corrections, introduced by the Bohm potential, play in modifying soliton dynamics.

The incorporation of the Bohm potential has profound effects on the dynamics of chiral solitons in the system. This quantum correction term modifies both the dispersion and stability of solitonic solutions. We observed that as the coefficient of the Bohm potential (δ) increases, the system exhibits several notable changes in the nature of solitons: 1. Width and Amplitude Modulation: The Bohm potential causes significant changes in the width and amplitude of the solitons, which can be attributed to the additional quantum pressure effects. These variations depend on the strength of the potential and can lead to broader or narrower solitons. 2. Formation of Multi-Peak Solitons: For larger values of the Bohm potential coefficient, multi-peak solitons emerge. This phenomenon is indicative of complex interactions between the nonlinear effects and quantum pressure, leading to multi-modal waveforms that can be of interest in various quantum fluid models. 3. Chaotic Transitions: As we increase the Bohm potential beyond certain thresholds, the phase portraits reveal a transition to chaotic behavior. This indicates that the inclusion of quantum effects introduces a level of complexity that can destabilize previously stable solitonic solutions, leading to chaotic and unpredictable dynamics.

The bifurcation analysis presented in the paper has allowed us to identify critical transition points in the system, where solitonic solutions undergo significant qualitative changes. The phase portraits, shown in Fig. 1, Fig. 2, provide clear visual representations of these bifurcations. We observed: 1. Stable Soliton Solutions: For small values of the Bohm potential, the system exhibits stable soliton solutions, as evidenced by closed orbits in the phase portraits. These stable solitons are characterized by localized waveforms that retain their shape during propagation. 2. Saddle-Node Bifurcations: In some regions of the parameter space, we identified saddle-node bifurcations, which are associated with transitions between stable solitons and dispersive wave solutions. This bifurcation behavior highlights the delicate balance between nonlinearity, dispersion, and quantum corrections in determining the system’s dynamics. 3. Dispersive and Chaotic Regimes: For larger values of the Bohm potential, the system undergoes transitions from stable localized solitons to dispersive waves and eventually to chaotic regimes. These transitions are marked by irregular trajectories in the phase portraits, pointing to the influence of quantum effects in generating complex soliton interactions.

The results of this study have several important theoretical and practical implications: 1. Quantum Hall Edge States: The interplay between nonlinearity and quantum corrections in the CNLSE model is directly relevant to understanding the dynamics of chiral edge states in the Quantum Hall Effect. This research sheds light on how quantum pressure influences the stability and interactions of these edge states, providing new insights into topologically protected quantum states. 2. Optical Solitons: The findings regarding soliton dynamics in the presence of the Bohm potential have implications for optical solitons in nonlinear media. By manipulating the Bohm potential coefficient, one could control the soliton properties such as amplitude, width, and stability. This could have applications in optical communication systems, where the propagation of solitons plays a key role in maintaining signal integrity. 3. Bose-Einstein Condensates (BECs): The study also has potential implications for the dynamics of matter-wave solitons in Bose-Einstein condensates. The inclusion of quantum effects, similar to the Bohm potential, could be used to describe quantum hydrodynamics in BECs, where solitonic solutions are stabilized by quantum pressure.

While this study provides a comprehensive bifurcation analysis and phase portrait study of chiral solitons with Bohm potential, there are several directions for future research: 1. Numerical Simulations: To validate the theoretical predictions, numerical simulations of the CNLSE with Bohm potential can be carried out. These simulations could provide more detailed insights into the behavior of solitons in regimes where analytical methods become challenging. 2. Higher-Dimensional Models: The current analysis is limited to one-dimensional systems. Extending this work to higher-dimensional settings, where the dynamics of chiral solitons may be even more complex, could provide valuable insights into multi-dimensional quantum fluid systems. 3. Experimental Realization: Experimental setups, such as those involving ultracold atomic gases or optical waveguides, could potentially realize the effects observed in this study. Future experimental investigations could help confirm the theoretical predictions and further explore the interplay between nonlinearity and quantum corrections in soliton dynamics.

This paper has presented a detailed analysis of chiral solitons governed by the chiral nonlinear Schrödinger equation (CNLSE) with the inclusion of a Bohm potential. The Bohm potential introduces significant quantum corrections that substantially modify soliton dynamics, resulting in phenomena such as multi-peak structures, amplitude modulation, and transitions toward chaotic behavior. Through bifurcation analysis and phase-portrait investigations, we have systematically identified the key stability properties and dynamical transitions within the system.

Beyond providing explicit analytical solutions, the present analysis clarifies the underlying bifurcation mechanisms that govern chiral soliton formation in the presence of Bohm-type quantum corrections. These findings fill an important gap in the theoretical framework describing nonlinear edge and guided modes in quantum and optical systems. The results have far-reaching implications for understanding nonlinear wave behavior in quantum fluids, with potential applications spanning quantum Hall edge transport, optical soliton propagation in chiral waveguides, and matter-wave excitations in Bose–Einstein condensates. The study thus offers a unified theoretical foundation and serves as a reference for future analytical, numerical, and experimental explorations of nonlinear and quantum-corrected soliton dynamics.

## Limitations

‘Not applicable’.

## CRediT authorship contribution statement

**Lu Tang:** Methodology, Formal analysis, Writing – original draft. **Yakup Yildirim:** Data curation, Investigation, Writing – review & editing, Visualization. **Ahmed H. Arnous:** Validation, Writing – review & editing. **Ahmed Shaker Mahmood:** Formal analysis, Writing – review & editing. **Ibrahim Zeghaiton Chaloob:** Resources, Writing – review & editing. **Anjan Biswas:** Conceptualization, Methodology, Writing – original draft, Funding acquisition.

## Declaration of competing interest

The authors declare that they have no known competing financial interests or personal relationships that could have appeared to influence the work reported in this paper.

## Data Availability

No data was used for the research described in the article.
